# Single-walled carbon nanotube interactions with HeLa cells

**DOI:** 10.1186/1477-3155-5-8

**Published:** 2007-10-23

**Authors:** Hadi N Yehia, Rockford K Draper, Carole Mikoryak, Erin Kate Walker, Pooja Bajaj, Inga H Musselman, Meredith C Daigrepont, Gregg R Dieckmann, Paul Pantano

**Affiliations:** 1 Department of Chemistry, The University of Texas at Dallas, Richardson, TX 75080, USA; 2 NanoTech Institute, The University of Texas at Dallas, Richardson, TX 75080, USA; 3 Department of Molecular & Cell Biology, The University of Texas at Dallas, Richardson, TX 75080, USA

## Abstract

This work concerns exposing cultured human epithelial-like HeLa cells to single-walled carbon nanotubes (SWNTs) dispersed in cell culture media supplemented with serum. First, the as-received CoMoCAT SWNT-containing powder was characterized using scanning electron microscopy and thermal gravimetric analyses. Characterizations of the purified dispersions, termed DM-SWNTs, involved atomic force microscopy, inductively coupled plasma – mass spectrometry, and absorption and Raman spectroscopies. Confocal microRaman spectroscopy was used to demonstrate that DM-SWNTs were taken up by HeLa cells in a time- and temperature-dependent fashion. Transmission electron microscopy revealed SWNT-like material in intracellular vacuoles. The morphologies and growth rates of HeLa cells exposed to DM-SWNTs were statistically similar to control cells over the course of 4 d. Finally, flow cytometry was used to show that the fluorescence from MitoSOX™ Red, a selective indicator of superoxide in mitochondria, was statistically similar in both control cells and cells incubated in DM-SWNTs. The combined results indicate that under our sample preparation protocols and assay conditions, CoMoCAT DM-SWNT dispersions are not inherently cytotoxic to HeLa cells. We conclude with recommendations for improving the accuracy and comparability of carbon nanotube (CNT) cytotoxicity reports.

## Background

The structural and electronic properties of SWNTs lend themselves to a variety of biomedical applications involving the detection and treatment of diseases, most notably cancer [1-6]. For example, the structural change in DNA upon shifting from the B to Z conformation sufficiently perturbs the electronic structure of SWNTs such that the change can be detected optically from living cells that have taken up DNA-SWNT complexes [7]. This and other works demonstrate how CNTs can be used as sensors within living cells [8,9]. In another example, exposing cells containing SWNTs to near infrared radiation kills the cells due to the efficient optical-to-thermal energy conversion of SWNTs, demonstrating that they can potentially be used in targeted cancer therapies to eliminate cancer cells [10]. Finally, there are a number of reports that CNTs facilitate the transport of bound oligonucleotides, peptides, and proteins across the plasma membrane [1,11-19]. However, despite these and other intracellular applications not listed here, there remain technical challenges towards realizing the potential benefits of CNTs in biomedicine. Namely, CNTs are extremely hydrophobic, bundle together, and are insoluble in water.

Two approaches have been used to modify the hydrophobic surface of CNTs to make them water soluble and biocompatible. The first has been to debundle and disperse CNTs in aqueous solution by covalently attaching water soluble substances to the CNT surface, and the second has involved the noncovalent association of material to the CNT surface [20-26]. In both approaches, a wide variety of organic adducts and biological materials have been used successfully including oligonucleotides [7,9,10,15,17,18,27-40], peptides [14,19,41-52], proteins [8,11-13,16,53-59] (most notably, bovine serum albumin (BSA) [60-63]), an assortment of polymers [64], and various cell culture media formulations [19,43,65-72]. While covalently attaching material to CNTs is advantageous for many applications, one serious drawback is that the covalent attachment introduces defects in the surface of the CNTs that often interfere with the electronic and optical properties that make CNTs so useful.

Beyond CNT dispersal, another challenge in the field is assessing whether CNTs are inherently cytotoxic [73-80]. At present, there are roughly as many publications reporting no apparent cytotoxicity [10,12-14,16-19,65-67,71,81-87], as there are reporting varying degrees of significant cytotoxicity [68-70,72,88-95]. Two major considerations in this area are how the CNTs are presented to the organism and the purity and concentration of the CNTs. For example, pulmonary toxicity of SWNTs has been established when large doses of dry, unpurified SWNTs have been blown into the lungs of rats [89,90,96]. This method of presentation is not relevant to the small measured doses of CNTs that would be used in chemotherapy and drug delivery. In fact, the biodistribution of chemically modified SWNTs injected into mice or rabbits was studied recently, and the CNTs were reported to be cleared rapidly with no evidence of toxicity [85,97,98]. CNT purity is also absolutely crucial. Many CNT syntheses use metal catalysts that are known to be toxic. Such impurities, and other carbonaceous impurities, must be removed from the samples in order to reach conclusions about inherent CNT toxicity, and it is not always clear from the published reports that they have been removed. Finally, many accounts of CNT toxicity have used MTT (3-(4,5-dimethyl-2-thiazolyl)-2,5-diphenyl-2H-tetrazolium bromide) as a reporter of cell viability, and it was recently shown by Worle-Knirsch et al. that MTT itself binds to CNTs (quenching its fluorescence) and thereby introducing uncertainty in this assessment of toxicity [65]. In summary, while the question of whether CNTs have long-term toxicity in biomedical applications requires further research, early reports raising the alarm of toxicity in model cell culture systems have not been adequately verified.

Recently, our group reported that HiPco SWNTs, dispersed in a peptide solution or in media supplemented with serum, were taken up by HeLa cells in a time- and temperature-dependent fashion and did not affect the HeLa cell growth rate, evidence that the SWNTs inside cells were not toxic under these conditions [19]. This work also demonstrated that our dispersion preparation protocol (involving probe sonication and multiple centrifugations) was effective in removing metals from the raw, as-received SWNT-containing powder. Herein, we present the characterizations of an as-received CoMoCAT SWNT-containing powder using thermal gravimetric analysis (TGA) and scanning electron microscopy (SEM), and of SWNTs dispersed in Dulbecco's modified Eagle medium (DMEM) supplemented with fetal bovine serum (FBS) using atomic force microscopy (AFM), inductively coupled plasma – mass spectrometry (ICP-MS), and absorption and Raman spectroscopies. The resulting purified dispersions, termed DM-SWNTs, are next shown to have no effect upon the morphologies and growth rates of HeLa cells – a thoroughly characterized human epithelial-like cell line. Using confocal microRaman spectroscopy, it is shown that DM-SWNTs were taken up by cells in a time- and temperature-dependent fashion. Evaluation of the distribution of intracellular DM-SWNTs was performed using transmission electron microscopy (TEM) which revealed SWNT-like material in vacuoles. Finally, intracellular superoxide dynamics of cells exposed to DM-SWNTs were evaluated using fluorescence-based flow cytometry and MitoSOX™ Red – a selective indicator of superoxide in mitochondria. The MitoSOX™ Red fluorescence detected from control cells was statistically similar to that observed for cells incubated in DM-SWNT dispersions. The combined results indicate that under our sample preparation protocols and assay conditions, CoMoCAT DM-SWNTs are not inherently cytotoxic to HeLa cells.

## Results and Discussion

### Characterizations of the as-received SWNT-containing powder

#### Microscopic analyses

**Figure 1:**
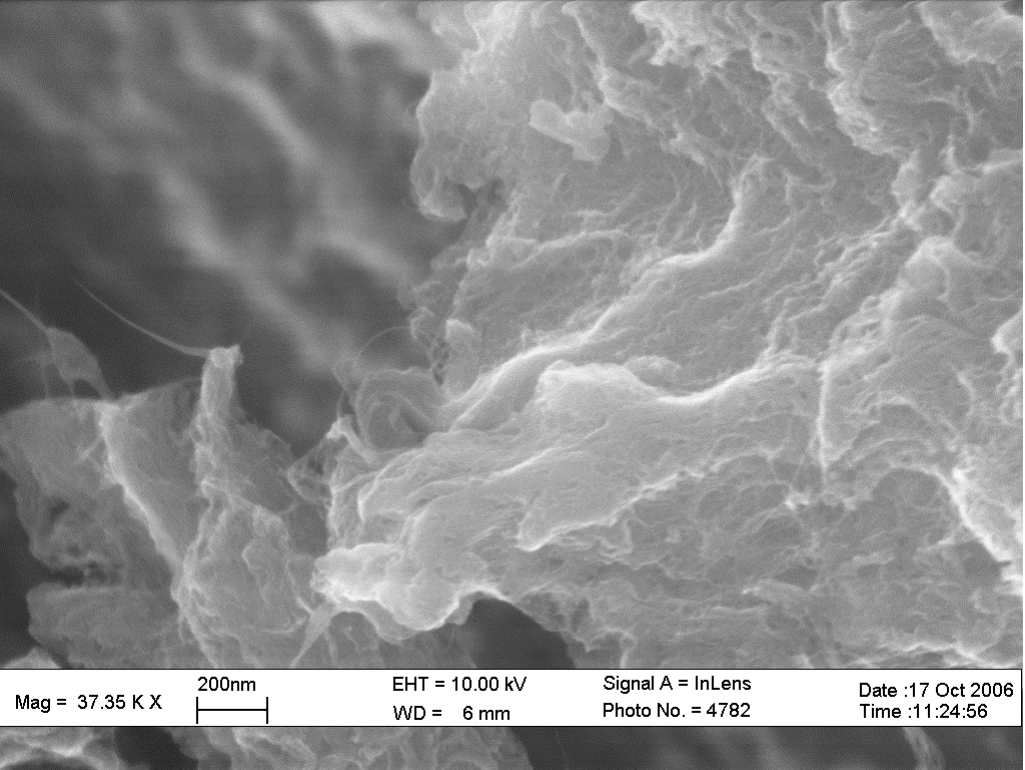
SEM image of the as-received CoMoCAT SWNT-containing powder on carbon black tape without a conductive coating.

The CoMoCAT method of SWNT synthesis involves a bimetallic Co-Mo catalyst supported on a silicon dioxide substrate [99-103]. The purification procedure includes removal of amorphous carbon by low-temperature oxidation, removal of the SiO_2 _substrate with HF, and removal of metals by HCl. The final product, a SWNT-containing powder, is rinsed with deionized water until its pH is neutral [104]. Visible microscopic examination of the lot used in this work revealed that the fine, black, fluffy powder comprised irregularly shaped particles with dimensions ranging from 5–50 μm. SEM revealed that the majority of these particles comprised tightly entangled networks of SWNTs, similar to those observed by Resasco and co-workers [105], and that these networks comprised small bundles of SWNTs with 5–20 nm diameters (Figure 1).

#### Thermal gravimetric analyses

**Figure 2:**
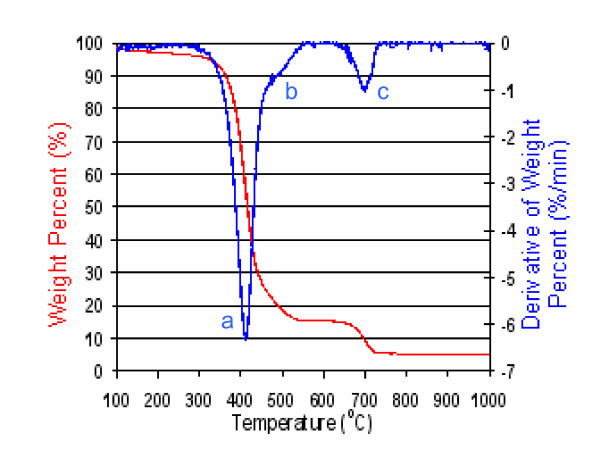
Weight percent and derivative of weight percent curves for the thermal gravimetric analysis of the as-received CoMoCAT SWNT-containing powder in oxygen.

TGA of the as-received SWNT-containing powder was performed to assess the powder's composition with respect to metals, SWNTs, and non-tubular carbon (NTC) species such as amorphous carbons, fullerenes, carbides, graphitic nanoparticles, etc. TGA measurements of the SWNT-containing powder were performed under the assumption that upon heating to 1000°C in O_2_, all carbon and metals were converted to their corresponding oxides, and that the presence of other trace elements could introduce small errors to calculated metal contents [106]. Figure 2 shows the weight percent decrease as a function of temperature (red trace) and the first derivative of the weight percent curve (blue trace) for the as-received SWNT-containing powder. The identities of the components corresponding to the three main peaks observed in the derivative plot were determined in experiments whereby the residues in the TGA pan were recovered and analyzed by Raman spectroscopy and/or XPS before, during, and after peak onset. In brief, peak *'a' *at ~410°C was determined to comprise SWNTs based on the appearance of a strong G-band – a Raman resonance uniquely associated with SWNTs. The oxidation temperature of the SWNTs ranged between 375–450°C and was consistent with the oxidation temperature of CoMoCAT SWNTs observed by Resasco and co-workers [105]. Peak *'b' *at ~505°C was determined to comprise NTCs based on the disappearance of the G-band and an increase of the D-band – a Raman resonance uniquely associated with miscellaneous forms of disordered carbon. Peak *'c' *at ~700°C, 9% weight loss, was determined to comprise MoO_3 _by XPS and was supported by the ~700°C sublimation temperature of MoO_3_. XPS experiments also ruled out the presence of residual SiO_2 _in the as-received SWNT-containing powder. The remaining 5% mass at 1000°C (Figure 2, red trace) was considered to be oxidized metals of Co and Mo, most likely CoMoO_4 _and MoO_2_. In summary, the oxidized SWNT-containing powder was classified as comprising ~70% SWNTs, ~7% NTC, and ~14% oxidized metals.

### Characterizations of SWNT dispersions

#### Absorption spectroscopy

**Figure 3:**
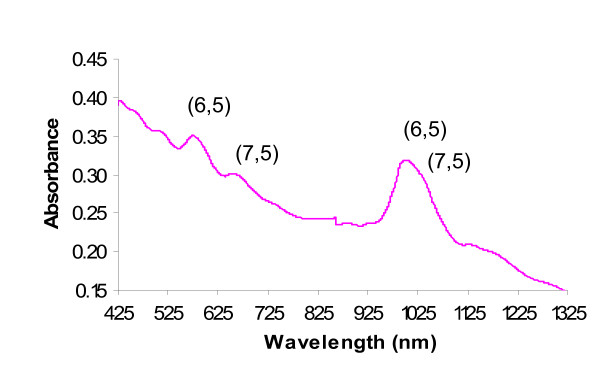
Background-corrected absorption spectrum of a CoMoCAT DM-SWNT dispersion prepared using a 10-min probe sonication and two 2-min centrifugations. The two main semi-conducting SWNT structures are denoted by their rollup vector integers (*n*, *m*), and the two absorptions at ~460 and ~515 nm represent metallic (*6*, *6*) and (*7*, *7*) nanotubes, respectively. The sharp feature at 861 nm is due to a grating and detector change associated with the spectrometer.

SWNT dispersions were prepared using a sonication and centrifugation protocol and DMEM supplemented with 5% FBS (DMEM/FBS). The resulting DM-SWNTs were homogeneous in appearance and could be stored for 30 d at 4°C before any SWNTs were observed to precipitate. The final concentration of SWNTs in DMEM/FBS was estimated to be ~50 μg/mL (Additional File 1) and SWNT lengths were estimated to be 100–400 nm (Additional File 2). Figure 3 shows the absorption spectrum of a representative DM-SWNT dispersion. The observed spectral profiles of DM-SWNTs were similar to the spectra of CoMoCAT SWNTs dispersed in sodium dodecyl sulfate (SDS) as prepared by Resasco and co-workers [103] and Stupp and co-workers [41], where the two predominant semi-conducting SWNT types present were (6,5) and (7,5) tubes with an average diameter of 0.8 nm. Specifically, the DM-SWNT peak observed at ~569 nm corresponds to the S_22 _optical transition of (6,5) tubes, the shoulder observed at ~587 nm corresponds to the S_22 _optical transition of (8,4) tubes, the peak observed at ~652 nm corresponds to the S_22 _optical transitions of (7,5) tubes at 644 nm and (7,6) tubes at 647 nm, the broad peak at ~1011 nm corresponds to S_11 _optical transitions of (6,5) tubes at 975 nm and (7,5) tubes at 1025, and the peak at ~1120 nm corresponds to the S_11 _optical transitions of (8,4) tubes at 1113 nm and (7,6) tubes at 1122 nm, which are all in accordance with spectroscopic assignments by Bachilo et al. [102]. In summary, the data indicate that CoMoCAT SWNTs dispersed in media supplemented with serum retain their optical transitions between van Hove singularities in the electronic density of states.

#### Raman spectroscopy

**Figure 4:**
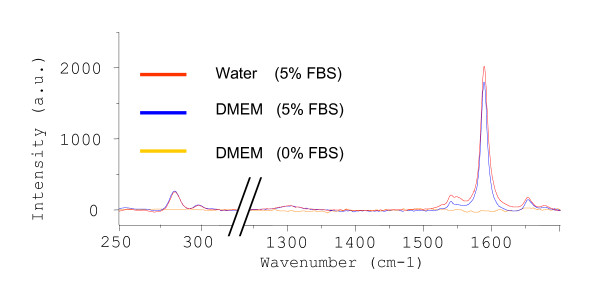
Raman spectra acquired from CoMoCAT SWNT dispersions (10-min probe sonication and two 2-min centrifugations) prepared in various solutions (water or DMEM ± FBS); all spectra were normalized to the same intensity scale.

Confocal microRaman spectrometer acquisition methods and the interpretation of the Raman spectra of various SWNT dispersions prepared using our sonication and centrifugation protocol have been detailed previously [19,45,47,49]. A representative Raman spectrum for a DM-SWNT dispersion is shown in Figure 4 (blue spectrum; DMEM + 5% FBS). The spectrum clearly shows a number of well characterized SWNT resonances [100,107,108], in particular, two predominant radial breathing modes at ~281 and ~301 cm^-1^, the D-band at ~1303 cm^-1^, and the G-band in the 1550–1610 cm^-1 ^region. Control spectra of DMEM/FBS without SWNTs did not display detectable resonances under these operating conditions (data not shown). Spectrometer stability was assessed by monitoring the reproducibility of the G-band peak intensity at ~1590 cm^-1 ^since it is the most prominent Raman peak indicative of intrinsic SWNT features [109]. In brief, the relative standard deviation (RSD) of G-band peak intensities acquired from the same region of a SWNT dispersion was <1%, the RSD of G-band peak intensities acquired from four different regions of a SWNT dispersion was <10%, and the correlation coefficient for the linear relationship between the G-band peak intensity and relative SWNT concentration was 0.982 (Figures S3-S5 in Additional File 3). In summary, the data indicates that the FBS components coating the SWNTs did not significantly affect the G-band profile of SWNTs dispersed in this fashion, which is in agreement with previous reports using non-covalently modified SWNTs dispersed in aqueous solutions of peptides [19,41,45] and proteins [53,62].

While a variety of cell types have been cultured in pristine or functionalized CNTs solubilized in various growth media formulations [19,43,65-72], only a few of these reports have emphasized the important role that the added serum plays. The importance of FBS in dispersing SWNTs is evident in the series of Raman spectra shown in Figure 4. In brief, DMEM comprises inorganic salts, amino acids, buffers, vitamins, and minerals with the three major components being glucose (4500 mg/L), sodium bicarbonate (3700 mg/L), and sodium chloride (6400 mg/L). FBS is also a multi-component mixture comprising many low and high molecular weight substances. The major dissolved substances are proteins, lipids, steroid hormones, minerals, and metabolites. The most notable FBS components known to solubilize CNTs are BSA and phospholipids [10,53,58]. Using our sonication and centrifugation procedure, DMEM without FBS did not support SWNT dispersion as observed by the lack of detectable SWNT Raman resonances (Figure 4; gold spectrum). Conversely, aqueous 5% FBS solutions (without DMEM) were quite effective in dispersing SWNTs (Figure 4; red spectrum).

#### ICP-MS analyses

Previous elemental analyses of peptide-coated SWNT dispersions prepared using our sonication and centrifugation protocol revealed only trace amounts of metal catalysts even though the as-received HiPco SWNT-containing powder contained ~32% metals by weight [19]. Herein, we further characterize this protocol's ability to effectively remove toxic materials by analyzing CoMoCAT DM-SWNT dispersions. CoMoCAT SWNTs are made from a process that uses Co and Mo as catalysts rather than Fe, and thus, exposing CoMoCAT SWNTs to cells avoids the known cellular toxicity that Fe can impact to a CNT preparation [66,70]. The ICP-MS analyses of DM-SWNT dispersions revealed 6.64 ppm Mo and 1.55 ppm Co, and that the only Fe in our DM-SWNT dispersions was from the 0.10 ppm ferric nitrate in DMEM. For comparative purposes in the absence of EC50 values for dispersed SWNTs, the metal levels observed in the DM-SWNT dispersions were well below the 90 ppm EC50 of mammalian stem cells exposed to 30-nm MoO_3 _particles (as determined by MTS assays) [110], and the 19 ppm EC50 of murine fibroblasts exposed to Co (as determined by MTT assays) [111]. Since >99% of the Mo and Co present in the as-received SWNT-containing powder was not detected in the DM-SWNT dispersions (relative to oxidized metal levels from the TGA of the SWNT-containing powder), these data again demonstrate that our sonication and centrifugation protocol is an effective method for removing the heavier metal-containing SWNTs and bundles. Such results are important to note since it has not been made clear in all previous published reports of cells being exposed to CNTs if such metal-removing measures were implemented before the CNT cytotoxicity was assessed.

DM-SWNTs were additionally analyzed for the presence of Ti since it is possible that this metal could be introduced through the use of Ti-coated probe sonicator tips. ICP-MS analyses of DM-SWNT dispersions prepared using a probe tip that had been used for >20 non-continuous hours revealed 0.15 ppm Ti. For comparison, this level is well below the 250 ppm EC50 of rat liver cells exposed to 40-nm TiO_2 _particles (as determined by MTT assays) [112]. To our knowledge, this is the first report of such an analysis amongst the previous reports of cells exposed to SWNT dispersions prepared using probe tip sonication.

### The uptake of DM-SWNTs by living cells

The main analytical approaches for assessing the presence of CNTs in cells and tissue have been optical [1,14,65,67,72,83,88,90,93,113], electron [11,15,17,37,68-70,89,114], and fluorescence [10,12-14,16,18,43,64,82,84,86] microscopies. While optical microscopy is ideally suited for live-cell analyses, this label-free technique lacks the specificity to unambiguously identify material observed in cells as CNTs. Electron microscopy offers high spatial resolution imaging of CNTs but is limited to slices of cells that have been fixed; multi-walled CNTs can be unmistakably identified in cells with this technique. In live-cell fluorescence microscopy, the detection of CNTs is indirect (i.e., it is through the detection of a visible fluorescent dye that is (non)covalently attached to the CNT or to molecules coating the CNT). Recently, direct and label-free mapping of CNTs inside living cells has been demonstrated using the intrinsic near-infrared fluorescence [7,9,81] or Raman scattering [9] of CNTs themselves.

#### Confocal microRaman spectroscopy of HeLa cells

**Figure 5:**
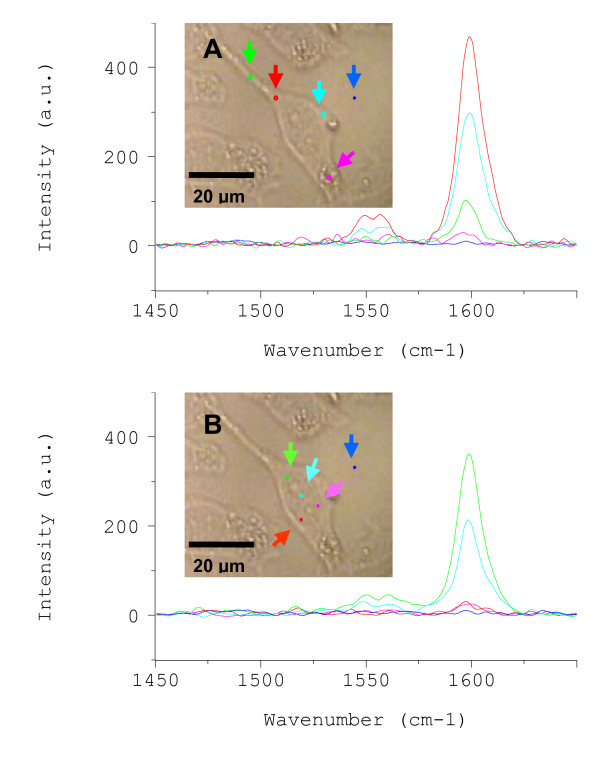
Raman spectra acquired from cytoplasmic **(A) **and nuclear **(B) **regions of the same live HeLa cell that was incubated at 37°C for 60 h in a CoMoCAT DM-SWNT dispersion. The colored arrows in the optical micrographs denote the specific regions of the HeLa cell where spectra were acquired; spectra were also acquired from cell-free regions of the culture dish ~5 μm away from the nearest cell (dark-blue arrows). All spectra were normalized to the same intensity scale.

Herein, the presence of CoMoCAT G-band intensities emanating from inside living cells incubated in DM-SWNT dispersions was evaluated using confocal microRaman spectroscopy. In the first series of experiments, cells were incubated in DM-SWNT dispersions for 60 h at 37°C. A representative transmitted white-light image of a single HeLa cell acquired through the Raman microscope is shown in Figure 5. Typical HeLa cells were observed to possess 10–30-μm widths and 40–70-μm lengths. The relatively large dimensions of HeLa cells, coupled with the 4-μm lateral resolution of the confocal microscope system, permitted Raman spectra to be acquired from distinct cellular regions [19]. For example, Figure 5 shows Raman spectra acquired from a cell that was incubated in a DM-SWNT dispersion. Intense G-band signals were observed from both cytoplasmic (Figure 5A) and nuclear (Figure 5B) regions. In the latter case, it should not be implied that SWNTs are in the nucleus because the detected G-band resonances could emanate from SWNTs located in the perinuclear region and/or in the cytoplasm immediately above or below the nucleus. Finally, control cells incubated in DMEM/FBS (without DM-SWNTs) had no detectable SWNT Raman signatures under these conditions (data not shown), and no SWNT resonances were detected from cell-free regions of the dish adjacent (≤5 μm) to cells (Figures 5A and 5B, dark blue spectra).

**Figure 6:**
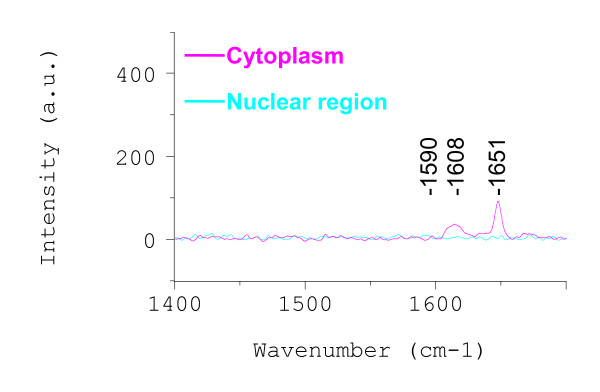
Raman spectra acquired from live HeLa cells incubated at 4°C in a CoMoCAT DM-SWNT dispersion; both spectra were normalized to the same intensity scale as that in Figure 5.

If the intense G-band signals emanated from DM-SWNTs inside cells, most likely the result of an active uptake process such as endocytosis, then the signals should be absent in cells exposed to DM-SWNTs at 4°C where energy-dependent uptake practically ceases. Figure 6 shows representative Raman spectra acquired from HeLa cells incubated in a DM-SWNT dispersion at 4°C. The peaks detected at ~1608 and 1651 cm^-1 ^in the spectrum acquired from the cytoplasm are presumed to emanate from proteinaceous material, as denoted by the amide-I band at 1650–1659 cm^-1 ^[115-117]. More importantly, the G-band intensities at ~1590 cm^-1 ^recorded from cytoplasmic and nuclear regions were 99.9% less than those recorded from cells incubated at 37°C (Figure 5). In summary, the lack of detectable G-band signals from HeLa cells incubated in DM-SWNT dispersion at 4°C indicates that HeLa cells do not uptake detectable levels of DM-SWNTs when their metabolic activity is low. In addition, the lack of G-band signals from cells incubated at 4°C indicates that there was negligible nonspecific adherence of SWNTs to HeLa cells (i.e., the rinsing procedures were sufficient to remove DM-SWNTs that were on the exterior surface of the plasma membrane).

#### Temporal evaluation of DM-SWNT uptake by HeLa cells

**Figure 7:**
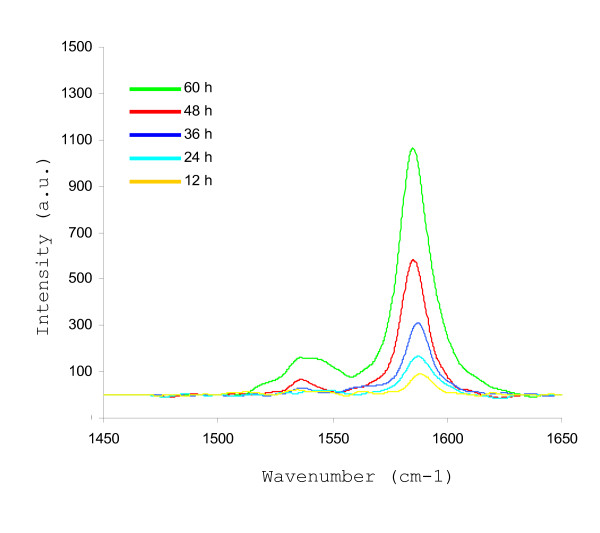
Representative Raman spectra acquired from five different live HeLa cells that were incubated at 37°C in CoMoCAT DM-SWNT dispersions for 12, 24, 36, 48, and 60 h. All spectra were normalized to the same intensity scale. The G-band intensities increased in a linear fashion (R^2 ^= 0.932) over the course of 12–60 h (n = 8 cells analyzed at each time point).

In another series of experiments, the time-dependence of DM-SWNT uptake was evaluated. First, the heterogeneous distribution of DM-SWNTs was taken into consideration. As shown in Figure 5, the G-band intensities detected from the cytoplasm ranged from 20 to 500 a.u. and those for the nuclear region ranged from 10 to 350 a.u. It was therefore decided to perform all time-dependent studies with the Raman laser focused on the center of a cell's nuclear region. This selection was influenced by our previous observations of SWNT accumulation around the nuclear region as revealed through confocal fluorescence imaging of HeLa cells exposed to SWNTs dispersed with a fluorescent-labeled peptide [118], and by Strano and co-workers through Raman spectral mapping of 3T3 cells exposed to SWNTs dispersed with DNA [9]. Figure 7 shows Raman spectra from HeLa cells that were incubated at 37°C in DM-SWNT dispersions for 12, 24, 36, 48, and 60 h. In all cases, the number of cells displaying detectable G-band signals increased as the DM-SWNT incubation time increased. Typically, the G-band intensities acquired from HeLa cells incubated in DM-SWNTs for 60 h was 90% greater than those detected at 12 h. Specifically, <10% of the cells analyzed after 12 h incubation displayed detectable G-band signals, while >90% of cells analyzed after 60 h displayed significant G-band signals (n = 40 cells analyzed). In summary, the combined Raman evidence indicated that the observed G-band intensities emanate from DM-SWNTs inside HeLa cells, and that the uptake of DM-SWNTs by HeLa cells is a time- and temperature-dependent process. While complete elucidation of the mechanism(s) of SWNT uptake by cells still requires further investigation, our results are consistent with the work of Dai and co-workers [12] and Cherukuri et al. [81] who have demonstrated that CNTs are transported inside cells via a temperature-dependent mechanism, and contrast the work of Bianco and co-workers who provide evidence that CNT uptake follows a temperature- and endocytosis-independent mechanism [14,37,43].

#### The intracellular distribution of DM-SWNTs

**Figure 8:**
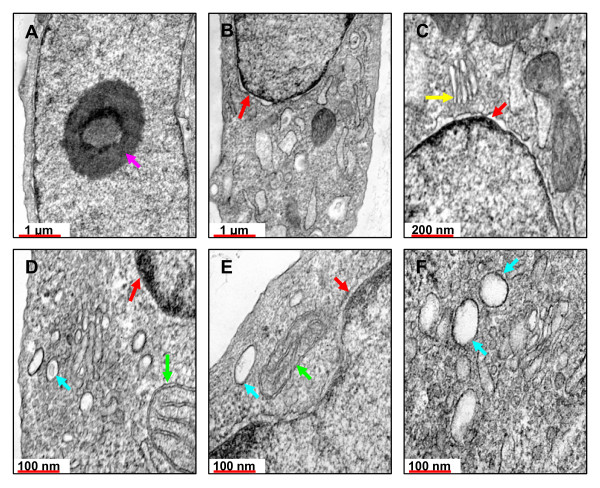
TEM micrographs of control HeLa cells that were incubated for 60 h at 37°C in DMEM/FBS (no DM-SWNTs). All slices were treated with uranyl acetate to stain membranes and lead citrate to stain the nuclear body. Colored arrows represent selected cell organelles: nuclei (red), mitochondria (green), Golgi bodies (yellow), vacuoles (blue), and the nucleolus (pink). Micrographs were normalized to the same grayscale as those in Figure 9.

**Figure 9:**
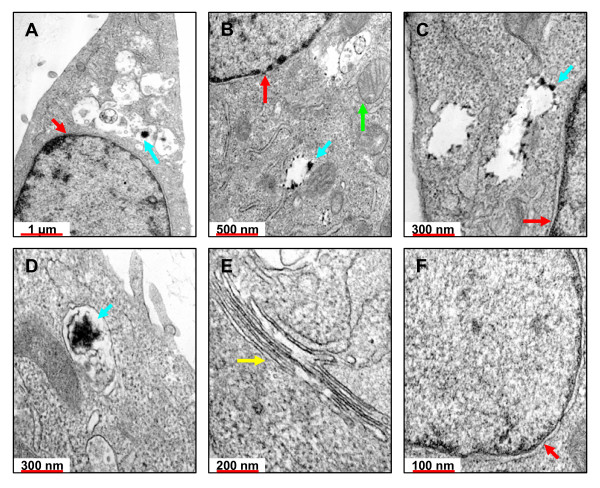
TEM micrographs of HeLa cells that were incubated for 60 h at 37°C in CoMoCAT DM-SWNTs. All slices were treated with uranyl acetate to stain membranes and lead citrate to stain the nuclear body. Colored arrows represent selected cell organelles: nuclei (red), mitochondria (green), Golgi bodies (yellow), and vacuoles (blue). Micrographs were normalized to the same grayscale as those in Figure 8.

TEM was used to examine the intracellular distribution of DM-SWNTs. Figures 8 and 9 show electron micrographs of HeLa cells incubated at 37°C for 60 h in DMEM/FBS (no SWNTs) or DM-SWNT dispersions, respectively. Colored arrows are used to denote the nucleolus and nucleus, vacuoles/vesicles, Golgi bodies, and mitochondria. In addition, it is important to note that all micrographs shown in Figures 8 and 9 were acquired from cells sliced in the plane of the nucleolus, as denoted by the low-magnification micrograph shown in Figure 8A. The first observation from the comparison of control and DM-SWNT treated cells was the lack of any SWNT-like structures visible in or associated with Golgi bodies (compare Figure 8C with 9E) and mitochondria (compare Figures 8D,E with 9B). The most striking observations between control (n = 8) and DM-SWNT treated (n = 10) cells was the appearance of dense black aggregated material in the cytoplasmic vacuoles of the DM-SWNT treated cells (Figures 9A–D) that was not observed in control cell vacuoles (Figures 8D–F). In the highest magnification view of these material-filled vacuoles (Figure 9D), the observed material displays black features with 5–20 nm diameters and apparent lengths of 50–300 nm, which is similar to the dimensions of CoMoCAT SWNTs in our dispersions. Such observations are consistent with those of Dai and co-workers who used confocal fluorescence microscopy to image the co-localization of SWNTs coated with a dye conjugate of avidin and the fluorescent endocytosis marker FM 4–64 [12,13].

Conclusive evidence of SWNT-like structures in the nucleus was not observed (compare Figures 8A–E to Figures 9A,B,C,F). This is important to note since there is presently no consensus regarding the ability of SWNTs to enter the cellular nucleus or the mechanism for their entry. For example, data that SWNTs have crossed the nuclear membrane has been presented by Bianco and co-workers using TEM and 300–1000-nm long peptide functionalized multi-walled CNTs [15], and Lu et al. using radioactive labels and ~400-nm long RNA-modified SWNTs [18]. In contrast, Strano and co-workers used confocal Raman imaging to observe DNA-coated SWNTs in the perinuclear zone of 3T3 cells, but not in the nuclear envelope [9]. In summary, amongst reports presenting high-resolution TEM images of cultured cells and tissue exposed to CNTs [11,15,17,37,68-70,89,114], it is apparent that large multi-walled CNTs can be unmistakably identified in cells by visual observation. The situation is more difficult when cells have been exposed to SWNTs. In most cases, the purported SWNT material appears as a single, dense black mass of material and there are few structural features observable on-scale with the expected diameters of individual/bundled SWNTs (± coatings). In fact, when SWNTs have been observed to be densely internalized in cell vacuoles [69], there are no observable differences between those TEM images and TEM images of cells exposed to fullerenes, which also display vacuoles densely filled with black material [119]. Clearly, the development of complementary analyses capable of identifying SWNT and NTC species in such images is warranted.

### Cell growth studies

**Figure 10:**
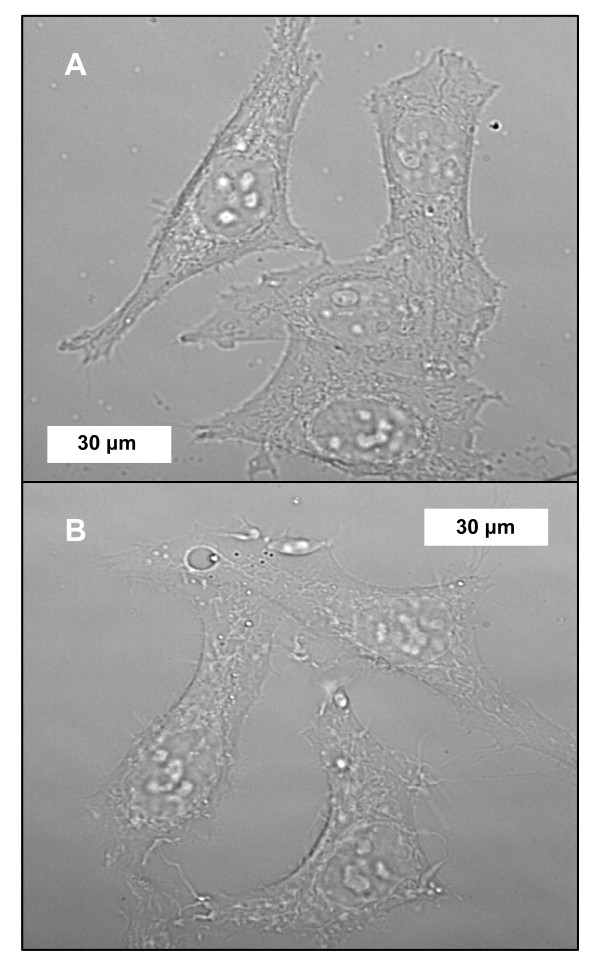
Representative differential image contrast (DIC) images of live HeLa cells incubated for 60 h at 37°C in DMEM/FBS **(A) **or CoMoCAT DM-SWNTs **(B)**.

**Figure 11:**
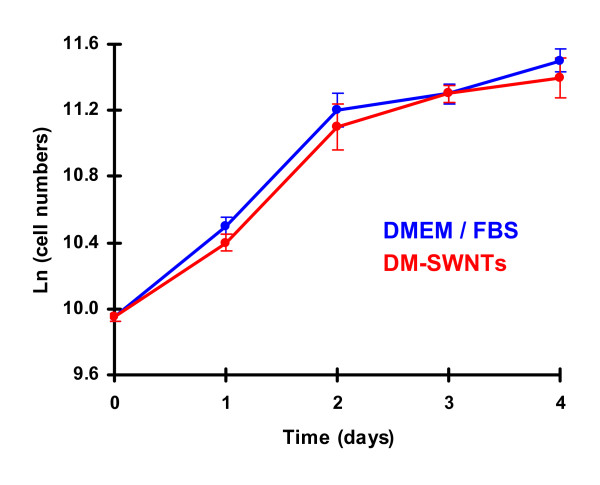
Growth curves for living HeLa cells incubated at 37°C for 4 d in DMEM/FBS or DM-SWNTs. The final concentration of SWNTs in DMEM/FBS was estimated to be ~50 μg/mL (Additional File 1) and SWNT lengths were estimated to be 100–400 nm (Additional File 2).

A crucial question amongst reports concerning the adherence and/or uptake of CNTs by cultured cells [1,7,9-18,37,43,65-73,75,77,78,81-84,86,88,91-94,113,114] is whether CNTs are toxic. Previously, we observed that the growth rates of HeLa cells incubated for 4 d in ~100 μg/mL HiPco SWNTs dispersed in a peptide solution or in media supplemented with serum were statistically similar to controls [19]. The evaluation of CoMoCAT DM-SWNTs also involved monitoring growth rates over the course of 4 d. First, there were no discernable differences in the morphologies of HeLa cells incubated in DM-SWNTs for 60 h (Figures 5 and 10B) relative to controls (Figure 10A; cells incubated in DMEM/FBS). Next, the growth rates of HeLa cells continuously exposed to DM-SWNTs were quantitated by calculating population double times (PDTs). A PDT is a measure of cell numbers at the early log growth phase and is used for comparisons of normal cell growth. PDTs were obtained from the slopes of the lines of a plot of the natural log of cell numbers versus time [120]. Figure 11 shows such a plot over a time period of 4 d for cells cultured in DM-SWNTs and control cells (DMEM/FBS only). For both samples, the respective number of HeLa cells counted on days 1, 2, 3, and 4 were statistically similar at a 95% confidence level. The control HeLa cell PDT was 27 h and was statistically similar to the PDT of 29 h observed with HeLa cells cultured in DM-SWNTs. In summary, the data from this sensitive test argue that our preparations and concentrations of purified CoMoCAT DM-SWNT dispersions do not affect HeLa cell growth rates.

### Intracellular superoxide dynamics of HeLa cells incubated in DM-SWNTs

As recommended by Worle-Knirsch et al., the presentation of CNT cytotoxicity results should include at least two or more independent test systems [65]. Therefore, in conjunction with morphology and growth rate studies, fluorescence-based flow cytometry was utilized to investigate whether the uptake of DM-SWNTs by HeLa cells increased the production of reactive oxygen species (ROS). In these series of experiments, HeLa cells were incubated in DM-SWNT dispersions and incubated with MitoSOX™ Red – a novel fluorescent indicator for the selective measurement of superoxide (O_2_^•-^) production in cells [121-123]. MitoSOX™ Red is a non-fluorescent, cell permeable dye that forms a highly fluorescent product upon oxidation. Owing to its lipophilic triphenyl phosphonium cation, MitoSOX™ Red is selectively targeted to mitochondria – the major source of ROS in cells – where it can be oxidized by superoxide before exhibiting red fluorescence upon binding to nucleic acids [123].

**Figure 12:**
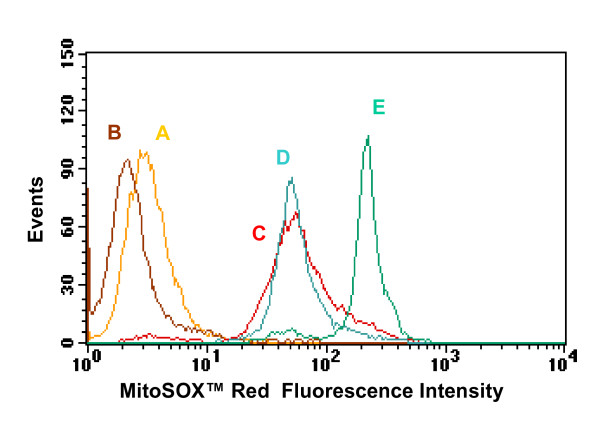
Flow cytometry analysis of intracellular MitoSOX™ Red fluorescence from live HeLa cells incubated at 37°C for 60 h in: **(A) **DMEM/FBS, **(B) **CoMoCAT DM-SWNTs, **(C) **DMEM/FBS + MitoSOX™ Red, **(D) **DM-SWNTs + MitoSOX™ Red, and **(E) **DMEM/FBS + MitoSOX™ Red + H_2_O_2_. The *x*-axis denotes the MitoSOX™ Red fluorescence detected in the 564–606 nm spectral region and the *y*-axis denotes the number of events recorded for each analysis.

In each fluorescence-based flow cytometry experiment, six different cell samples/controls were prepared and analyzed in triplicate with each individual trial representing the analysis of thousands of cells. Fluorescence microscopy was also used to validate that MitoSOX™ Red was distributed throughout the cytoplasms of cells, and that negligible dye leaked from the cells (data not shown). The first two flow cytometry control experiments involved measuring responses of cells incubated in DMEM/FBS without MitoSOX™ Red (± DM-SWNTs). These dye-free controls were prepared to establish background fluorescence levels of unstained HeLa cells (± DM-SWNTs) and are represented in the plot of events vs. MitoSOX™ Red fluorescence intensities as shown in Figures 12A &12B (and Figures S6A&B in Additional File 4). The means and standard deviations of fluorescence intensities from these two control experiments without MitoSOX™ Red were 3.07 ± 0.15 and 2.40 ± 0.44 a.u. for DMEM/FBS and DM-SWNT treated cells, respectively. Next, since it has recently been reported that binding of fluorescent viability dyes to CNTs can add uncertainty to cytotoxicity assessments [65], our series of experiments also included a comparison of responses from positive controls ± DM-SWNTs. Specifically, the responses of cells loaded with MitoSOX™ Red and exposed to 5 μmoles hydrogen peroxide were analyzed in the presence and absence of DM-SWNTs (Additional File 4; Figures S7A and S7B respectively). Both samples possessed statistically-similar fluorescence intensities indicating that SWNT quenching of the MitoSOX™ Red fluorescence was minimal.

Figures 12C &12D (and Figures S6C&D in Additional File 4) show representative responses of cells loaded with MitoSOX™ Red and incubated either in a DMEM/FBS control (no SWNTs) or in a DM-SWNT dispersion. The means and standard deviations of fluorescence intensities from these two experiments (51.0 ± 24.2 and 47.3 ± 22.1 a.u. for DMEM/FBS controls and DM-SWNT treated cells, respectively) were statistically similar. For comparison, the mean fluorescence intensities from the positive control shown in Figure 12E (and Figure S6E in Additional File 4) was ~7-fold greater (343 ± 101 a.u.). These results are akin to the results of Shvedova and co-workers who observed interesting relationships between the metal content of SWNTs and the iron-induced intracellular production of ROS. In brief, SWNTs containing 26.0 wt% Fe stimulated significant production of hydroxyl radicals by RAW 264.7 macrophages (vs. purified SWNTs containing 0.23 wt % Fe as detected by electron paramagnetic resonance spin-trapping assays), while fluorescence analyses with dihydroethidium incubated macrophages revealed similar superoxide and nitric oxides levels for both cells exposed to the Fe-containing SWNTs or purified SWNTs [66]. Nonetheless, while superoxide is just one of many potential reactive oxygen and nitrogen species, and while Co, Mo, Ti, and Fe are just four types of potential metal impurities, these data suggest that our preparations and concentrations of purified DM-SWNTs do not increase the concentrations of mitochondrial superoxide in HeLa cells under these culture conditions.

## Conclusion

Herein, CoMoCAT SWNT-containing powders and DM-SWNT dispersions were characterized using AFM, ICP-MS, SEM, TGA, and absorption and Raman spectroscopies. Confocal micoRaman spectroscopy was utilized to determine that DM-SWNTs entered HeLa cells in a time- and temperature-dependent fashion. TEM revealed SWNT-like material in intracellular vacuoles. Flow cytometry showed that the fluorescence from MitoSOX™ Red, a selective indicator of superoxide in mitochondria, in control cells was statistically similar to that observed for cells incubated in DM-SWNTs. The morphologies and growth rates of HeLa cells exposed to DM-SWNTs were statistically similar to control cells over the course of 4 d. The combined results indicate that, using our sample preparation protocols (i.e., probe tip sonication followed by two centrifugations), and under our assay conditions (i.e., SWNT types, coatings, dimensions, concentrations, impurity types and amounts, and cellular exposure times), CoMoCAT DM-SWNT dispersions are not inherently cytotoxic to HeLa cells. Finally, the importance of thoroughly characterizing CNT materials before offering a CNT cytotoxicity assessment can not be over emphasized. We support the development of (i) standardized CNT sample preparation protocols, reference materials, and characterization methodologies, (ii) standardized methods for assessing whether CNTs are taken up by and/or adsorbed to cells, and (iii) a series of proven cell vitality assay conditions. Such measures are imperative to improve the accuracy and comparability of CNT cytotoxicity reports.

## Methods

### Media and solutions

Dulbecco's modified Eagle medium (DMEM) was purchased from Irvine Scientific and was supplemented with 3700 mg/L sodium bicarbonate, 1% (v/v) penicillin, streptomycin, and amphotericin B (Sigma-Aldrich). Fetal bovine serum (FBS) was obtained from HyClone. Phosphate buffered saline (PBS; 8 mM phosphate, 150 mM NaCl, pH = 7.4) was sterilized by autoclaving at 120°C for 0.5 h. Deionized water (18.3 MΩ-cm) was obtained using a Nanopure Infinity water purification system (Barnstead). All other chemicals were of the highest quality available and were used as received.

### SWNT dispersions

All dispersions were prepared with CoMoCAT SWNTs (Product No. SP95-02-dry, Lot No. UT3-A001; SouthWest NanoTechnologies Inc.). The preparation of DM-SWNTs (i.e., SWNTs dispersed in DMEM supplemented with 5% (v/v) FBS (i.e., DMEM/FBS)) used a sonication/centrifugation protocol identical to that previously described by Chin et al. except that the centrifugation times were reduced [19]. Specifically, 1.0 mg of the as-received SWNT-containing powder was dispensed into an Eppendorf tube containing 1.0 mL of DMEM/FBS, vortexed for ~1 min, and probe sonicated for 10 min at 0°C. Probe-sonication was performed using a Branson 250 Sonifier, and the 2 mm diameter probe tip was placed one-third of the distance below the surface of the 1 mL suspension. The resulting black suspension was centrifuged in an Eppendorf 5417C centrifuge for 2 min at 16,000 *g *(14,000 RPM). The upper 75% of the supernatant was recovered without disturbing the sediment and placed in a clean tube before a second 2 min centrifugation at 16,000 *g *was performed. The upper 75% of the second supernatant was carefully recovered to afford a DM-SWNT dispersion. The preparation of aqueous dispersions in 0.15% (v/v) sodium dodecyl sulfate (SDS-SWNTs), 0.1% (v/v) TritonX-100 (TrX-SWNTs), or 5% (v/v) FBS (FBS-SWNTs) was identical to that described above except that DMEM/FBS was replaced by the corresponding surfactant or serum.

### Scanning electron microscopy

SEM was performed at 10 kV with a Zeiss-LEO Model 1530 variable pressure field effect scanning electron microscope. Samples of the as-received SWNT-containing powder were placed on a SEM mount with carbon black tape and analyzed without a conductive coating.

### Thermal gravimetric analysis

TGA was performed with a Perkin Elmer Pyris-1 thermal gravimetric analyzer equipped with a high temperature furnace and sample thermocouple. Samples (n = 3) of the as-received SWNT-containing powder were dried in air for 6 h at 100°C before being transferred into the platinum pan of the analyzer. The samples were heated from room temperature to 1000°C at 5°C/min in >99.9% O_2 _using a flow rate of 20 mL/min. A baseline was generated for each scan and baseline-subtracted thermograms were converted to weight percents. Thermal oxidation temperatures were identified by the peaks from the derivative of weight percent curve. Triplicate analyses yielded oxidation temperatures with a reproducibility of ± 2°C. The determination of a component's mass was performed by subtracting the weight percent lost between peak onset and end. In the case where two peaks overlapped (Figure 2, peaks *'a' *and *'b'*), the weight percent lost for the non-overlapping half of each peak was calculated and doubled. Validation of this approach was performed through a Gaussian peak fitting routine to determine the weight percent loss (i.e., the peak area) of each component; the reported masses from the two methods matched within ± 1%. The total mass of oxidized metal was reported as the sum of the mass from MoO_3 _(peak *'c'*) and the mass remaining at 1000°C. Triplicate analyses demonstrated mass accuracies of ± 0.2%. The initial weight loss ≤300°C was ~5%. While additional error could be attributed to weight gain by the oxidation of metals, the major source of error in reported weight percentages emanated from the fitting of peaks with components displaying overlapping oxidation temperatures.

### Absorption spectroscopy

The absorption spectra of DM-SWNTs were acquired using a dual-beam Perkin Elmer Lambda 900 UV-VIS-NIR spectrophotometer and were background-corrected using DMEM/FBS. Scans were performed from 400–861 nm with a scan speed of 125.00 nm/min and a 0.44-s integration time and from 861–1350 nm with a scan speed of 125.00 nm/min and a 0.48-s integration time. The instrument was wavelength calibrated on a quarterly basis using Holmium standards.

### Elemental analysis

Elemental analysis was performed using a ThermoElectron X-Series inductively coupled plasma mass spectrometer. Samples (100 μL of DMEM/FBS or DM-SWNTs) were acid digested using a protocol developed in association with PreciLab Inc. (Addison, TX). In brief, a solution of 25 μL of 37% HCl and 25 μL of 69% HNO_3 _was added to samples which were bath ultrasonicated for 20 min. Next, the samples were diluted with a 2% HNO_3 _blank to a total volume of 10 mL. All samples and standard solutions were sprayed into flowing argon and passed into the torch which was inductively heated to ~10,000°C. Ti and Co were calibrated using blank, 50-, 100-, and 250-ppt standard solutions, Mo was calibrated using blank, 250-, 1000-, and 5000-ppt standard solutions, and Fe was calibrated using blank, 0.25-, 1.0- and 5.0-ppb standard solutions.

### Primary cell culture

Human epithelial-like HeLa cells were obtained from the American Type Culture Collection and were cultured in 100 mm diameter polystyrene tissue culture dishes (Sarstedt) in DMEM/FBS containing 15 mg/L phenol red in an incubator at 37°C with 90% air and 10% CO_2_. Aseptic conditions were maintained at all times and media was changed every 2 d. Cells were passaged 1:10 every 4 d upon achieving ~80% confluence.

### Population doubling time assays

HeLa cells were plated into standard 24-well plates (~1 × 10^4 ^cells/well; ~20% coverage) in DMEM/FBS (buffered with 10 mM HEPES; no bicarbonate) and incubated in air at 37°C. After 24 h, the media was removed and replaced by a 400-μL aliquot of freshly prepared DM-SWNTs or fresh media (control). Each group of cells was incubated further in air at 37°C for 1–4 d. On each day, some HeLa cells were washed twice with 400 μL of sterile PBS and harvested with 100 μL of trypsin-EDTA solution (Irvine Scientific) for Coulter cell counting. Population doubling times (PDTs) were determined using the equation *PDT = ln (N/N*_*o*_*)/t*, where *N*_*o *_represents the initial cell number, *N *represents the final cell number, and *t *represents the time interval between *N*_*o *_and *N *[120]. Each group of cells was analyzed in triplicate; one-way ANOVA statistical analyses were performed at the 95% confidence level, where *p *< 0.05 was considered significant. Differential image contrast (DIC) images were acquired using a Nikon TE 2000-U inverted microscope and a 60×/1.4 *NA *APO-Plan oil-immersion objective.

### Confocal microRaman spectroscopy

All Raman spectra acquisition and sample preparation methods were similar to those described previously by Chin et al. [19]. Spectra were acquired utilizing a Horiba Jobin Yvon high-resolution LabRam Raman microscope system equipped with a 250-μm entrance slit and a 400-μm pinhole. The 633-nm laser excitation was provided by a Spectra-Physics model 127 helium-neon laser operating at 20 mW. The power density emanating from the 50×/0.5 *NA *LM-Plan objective was typically 3.4 mW as measured using a Newport model-1815C power meter with an 818 UV series photodetector. Wavenumber calibration was performed using the 520.5 cm^-1 ^line of a silicon wafer; the spectral resolution was ~1 cm^-1^.

Raman spectra of SWNT dispersions were acquired by placing them into 35 mm polylysine-coated glass bottom "imaging" dishes (MatTek). The acquisition time for a 250-cm^-1 ^spectral region was 10 s with a scan speed of 0.04 cm^-1^/s; all spectra were plotted as the average of three scans. For live-cell analyses, ~1 × 10^5 ^HeLa cells were seeded in imaging dishes with DMEM/FBS and incubated at 37°C in 90% air and 10% CO_2_. After 24 h, the media was removed and the HeLa cells were rinsed three times with sterile PBS. The cells were incubated further in air at 37°C (or 4°C) in 1 mL of either DMEM/FBS (control) or a freshly prepared DM-SWNT dispersion. Following the designated DM-SWNT incubation period (12–60 h), the cells were copiously rinsed at least three times with sterile PBS. After excess PBS was removed from the dish, 1 mL of fresh media was added and the dish was placed on the microscope stage for analysis at room temperature. Adherent cells were brought into focus by viewing transmitted white-light images obtained through a CCD video camera. The Raman acquisition time for a 250 cm^-1 ^spectral region was 45 s with a scan speed of 0.18 cm^-1^/s; all spectra were plotted as the average of three scans.

### Transmission electron microscopy

Live cells were incubated in a DM-SWNT dispersion (or a DMEM/FBS control) for 60 h as described above. After the final PBS rinsing, the cells were fixed using 2.5% glutaraldehyde in 0.1 M cacodylate buffer and embedded in agarose. Cell pellets were cut into small pieces, post-fixed with 1% osmium tetroxide, en-bloc stained with 1% uranyl acetate, dehydrated in a graded ethanol series, and embedded in EMbed-812 resin. Ultrathin (~100 nm) sections were cut on a LEICA EM UC6 ultramicrotome, post-stained with uranyl acetate and lead citrate, and viewed using the JEOL JEM-1200EX II electron microscope at the Molecular and Cellular Imaging Facility at The University of Texas Southwestern Medical Center.

### Flow cytometry

In all flow cytometry experiments, ~1.0 × 10^6 ^HeLa cells were seeded in imaging dishes with DMEM/FBS and incubated at 37°C in 90% air and 10% CO_2_. After 24 h, the media was removed, the cells were rinsed with sterile PBS, and the cells were incubated in fresh DMEM/FBS (control) or a DM-SWNT dispersion in air at 37°C for 60 h. In some cases, cells were rinsed at least three times with sterile PBS and loaded with a solution of MitoSOX™ Red (Invitrogen-Molecular Probes). Specifically, cells were incubated for 60 min at 37°C in a 10 μM MitoSOX™ Red solution prepared in 4:1 (v/v) DMEM/PBS. Next, cells were rinsed three times with PBS, harvested with 500 μL of trypsin-EDTA solution, centrifuged at 5000 RPM for 5 min, and resuspended in 3 mL of fresh 2% (v/v) FBS/PBS. Finally, cell suspensions were filtered through a 30-μm PreSeparation filter (Miltenyi Biotec). Fluorescence-based flow cytometry was performed using a Becton Dickinson FACSCalibur ^® ^flow cytometer equipped with a 488 nm laser. MitoSOX™ Red fluorescence (λ_Max _= 590 nm) was detected over the range of 564–606 nm and the background fluorescence was detected over the range of 515–545 nm. All quantitations were performed using CellQuest 7.5.3 software; in each experiment, well over 10,000 cells were analyzed.

## Competing interests

The author(s) declare that they have no competing interests.

## Authors' contributions

HNY performed the majority of the experiments and wrote the manuscript with PP. GRD, RKD, IHM, and PP designed the overall project and aided with data interpretations. CM ran the culturing facility and assisted with the interpretation of live cell data. EKW performed and interpreted the thermal gravimetric analyses. PB performed and interpreted the scanning probe analyses. MCD performed and interpreted the elemental analyses.

## Supplementary Material

Additional file 1 Supporting thermal gravimetric analysis data. Estimation of SWNT concentrations in DM-SWNT dispersions. Click here for file

Additional file 2 Supporting atomic force microscopy data. Atomic force microscopy of SWNT dispersions. Click here for file

Additional file 3 Supporting Raman spectroscopy data. Raman spectrometer reproducibility and calibration. Click here for file

Additional file 4 Supporting flow cytometry data. Event plots. Click here for file
