# Supplementary material for: Single-walled carbon nanotube interactions with HeLa cells
Source: J Nanobiotechnology. 2007 Oct 23;5:8. doi: 10.1186/1477-3155-5-8 (PMC2131758; doi:10.1186/1477-3155-5-8)
Supplement: Additional file 1 — Supporting thermal gravimetric analysis data. Estimation of SWNT concentrations in DM-SWNT dispersions. [file 1477-3155-5-8-S1.doc]

TGA of DM-SWNTs was performed to estimate the concentration of SWNTs present in these dispersions. The TGA protocol was identical to that described in the materials and methods section. The weight loss profiles between 100-1000 °C for DM-SWNT dispersions and controls (i.e., DMEM/FBS solutions without SWNTs) were essentially indistinguishable, as was to be expected for two, nearly-identical multicomponent mixtures. In brief, >50% of the weight of the DMEM/FBS mixture comprised species that burned <520 °C, and >40% of the weight comprised species that burned in the 770-920 °C temperature range.**^a^** Fortunately, distinct profiles were observed in the temperature range where SWNTs were oxidized (as determined from the TGA of the SWNT-containing powder shown in Figure 2). Figure S1 shows the derivative weight percent curve for DM-SWNTs (blue trace) and for DMEM/FBS (red trace). Ideally, one would simply subtract the weight loss observed in the 360-450 °C range of the DMEM/FBS sample from that of the DM-SWNTs to determine the weight loss due to SWNTs (Table S1, column 3). However, in comparing the weight loss profiles between DM-SWNTs and DMEM/FBS (Figure S1), it is apparent that a simple subtraction of weight losses would not be accurate since the two samples have different starting weights in the ~360-390 °C region. Therefore, the *percent-weight-lost* for DMEM/FBS and DM-SWNT samples were calculated and subtracted to yield a 0.94%-weight-lost that is primarily attributable to SWNTs (Table S1, column 4). Since 0.94% of the weight of dried DM-SWNTs (Table S1, column 2) corresponds to 0.050 mg, the concentration of SWNTs in the DM-SWNT dispersion is ~50 μg/mL.

**Figure S1**

Derivative of weight percent curve for the thermal gravimetric analysis of DM-SWNTs (blue trace) and DMEM/FBS (red trace). Both samples were dried in air for 6 h at 100 °C before being transferred to the analyzer. The samples were heated from room temperature to 1000 °C at 5 °C/min in >99.9% O_2_ using a flow rate of 20 mL/min.

**Table S1**

Calculations based on the TGA curves for the DM-SWNT and DMEM/FBS samples shown in Figure S1.

**^a^** Interestingly, there were no significant weight loss differences between DMEM/FBS and DM-SWNTs in the 600-770 °C temperature range. Since MoO_3_ was observed to oxidize at ~700 °C in the TGA of the SWNT-containing powder (Figure 2), this data provides additional evidence that our dispersion preparation protocol (involving probe sonication and multiple centrifugations) is effective in removing metal impurities.
