# Supplementary material for: Single-walled carbon nanotube interactions with HeLa cells
Source: J Nanobiotechnology. 2007 Oct 23;5:8. doi: 10.1186/1477-3155-5-8 (PMC2131758; doi:10.1186/1477-3155-5-8)
Supplement: Additional file 2 — Supporting atomic force microscopy data. Atomic force microscopy of SWNT dispersions. [file 1477-3155-5-8-S2.doc]

Atomic force microscopy (AFM) was employed to assess the dimensions of DM-SWNTs with the goal being the generation of dispersions enriched with short SWNTs that might be more readily taken up by cells. AFM images were acquired in air under ambient conditions with a Digital Instruments Nanoscope III MultiMode scanning probe microscope. Images were acquired in the TappingMode™ using cantilevers with 0.9 N m^-1^ force constants as described by Musselman and co-workers [46]. Samples were prepared by drop-casting a 10-μL aliquot of the SWNT dispersion onto freshly cleaved muscovite mica (Asheville-Schoonmaker Mica Co.); samples were also prepared by spin-casting the SWNT dispersion onto mica at 800 *g* for 30 s. All samples were dried at room temperature in a desiccator for 24 h prior to imaging.

Previously, using 10-min sonication times, aqueous dispersions of peptide-coated HiPco SWNTs (without DMEM/FBS) were observed via AFM to possess individual tubes and small bundles with diameters ranging from 1-2 nm and lengths ranging from 100-400 nm [19]. In the present case, using 10-min sonication times, attempts to image dispersions of CoMoCAT SWNTs prepared in DMEM/FBS and drop-cast onto mica were unsuccessful. In brief, the viewing of individual SWNTs was obscured by crystals from the high concentrations of sugars and salts in DMEM and proteinaceous material from the FBS (images not shown). Since DMEM alone did not support SWNT dispersion and FBS did (Figure 4), AFM was performed next on dispersions prepared in an aqueous 5% FBS solution drop-cast onto mica. In this case, a thick layer of proteinaceous material covered the mica surface and obscured the viewing of individual SWNTs (images not shown). However, when the same FBS-SWNT dispersion was spun-cast on mica, the AFM image (Figure S2A) revealed structures that possessed relatively short lengths (20-100 nm) and diameters on the order of 2-10 nm. Since control images of FBS solutions (without SWNTs) did not reveal any such structures, the structures in Figure S2A are indicative of heavily coated individual SWNTs and/or small SWNT bundles. Further evidence supporting the ability of our sonication and centrifugation procedure to afford individual and/or small bundles of short SWNTs was acquired using aqueous dispersions of SWNTs prepared in a customary surfactant. In these experiments, SDS was chosen for the analyses since it afforded aqueous dispersions with less emulsions relative to TritonX-100. As shown in Figure S2B, AFM images of drop-cast CoMoCAT SDS-SWNT dispersions on mica revealed individual SWNTs with diameters of 1-3 nm and lengths in the range of 100-400 nm (similar to our previous observations with peptide-coated HiPco SWNTs [19]). In summary, the acquisition of AFM images of SWNTs dispersed in a complex mixture with high concentrations of sugars, salts, and proteins, such as cell culture media supplemented with serum, is technically challenging due to the thick coatings. Nonetheless, our observations with FBS-SWNTs are akin to AFM images of SWNTs noncovalently dispersed in aqueous solutions containing a single biomolecule such as an oligonucleotide [10, 32, 33, 39], a lipid [82], or a protein [12, 54, 56], and aqueous dispersions of SWNTs covalently derivatized with protein [53, 57, 60-63].


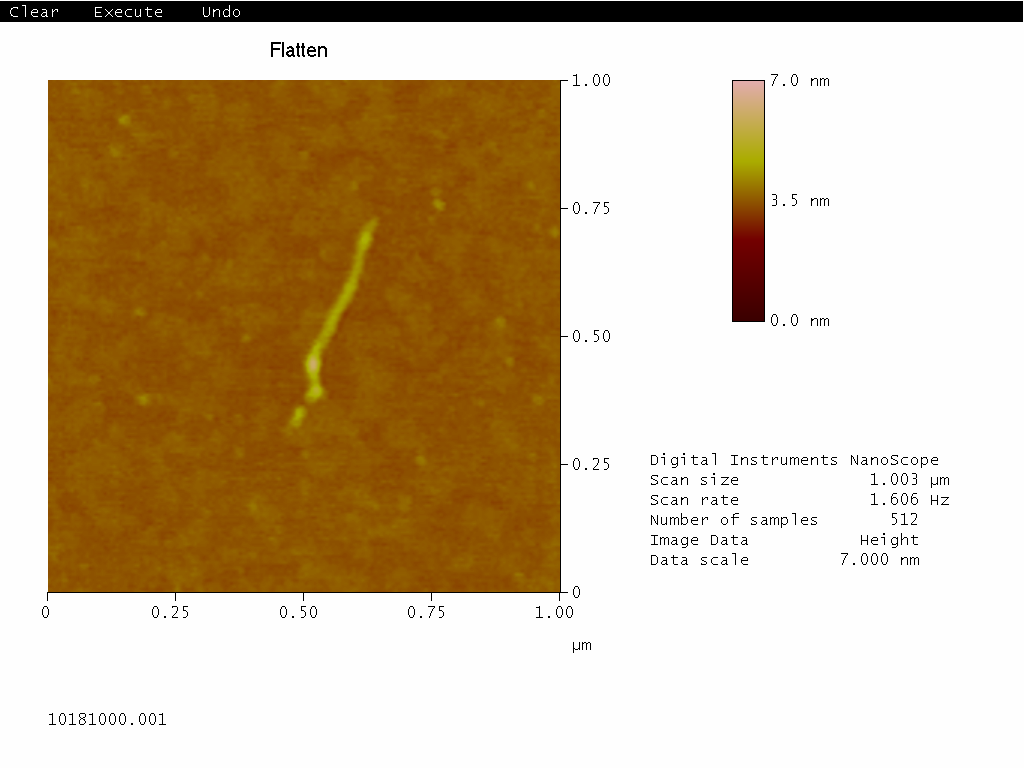


**200 nm**

**B**


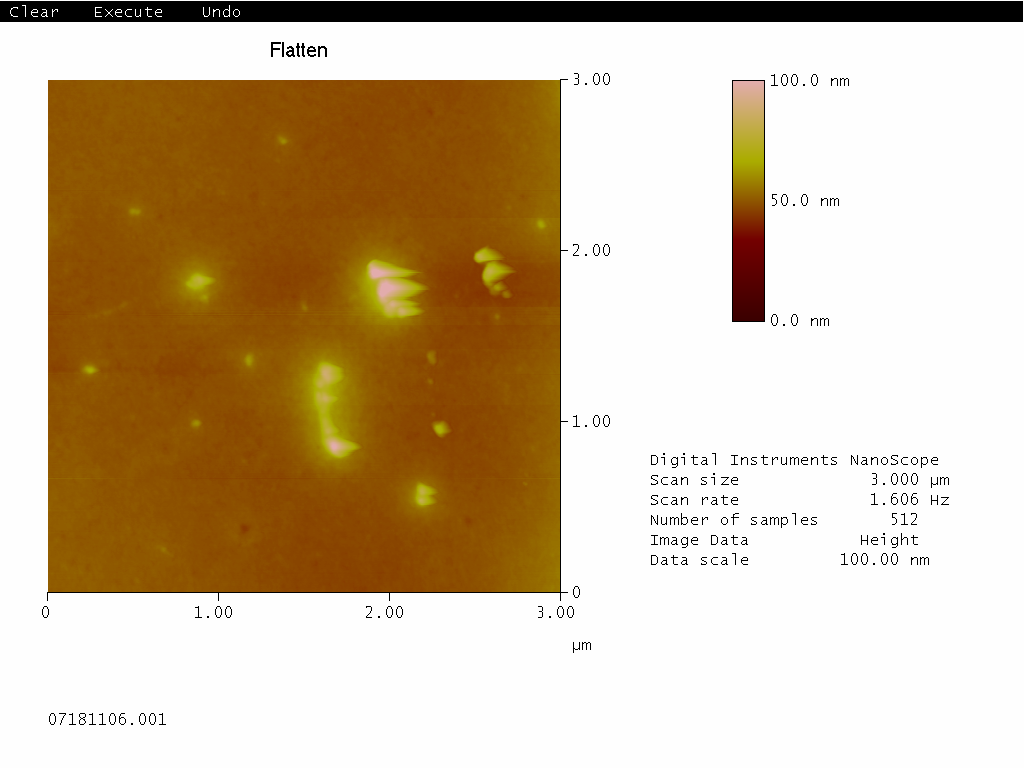


**200 nm**

**A**

**Figure S2**

AFM images of a FBS-SWNT dispersion spun-cast onto mica **(A)**, and a SDS-SWNT dispersion drop-cast onto mica **(B)**. Both CoMoCAT SWNT dispersions were probe sonicated for 10 min and centrifuged twice for 2 min.
