# Supplementary material for: Single-walled carbon nanotube interactions with HeLa cells
Source: J Nanobiotechnology. 2007 Oct 23;5:8. doi: 10.1186/1477-3155-5-8 (PMC2131758; doi:10.1186/1477-3155-5-8)
Supplement: Additional file 3 — Supporting Raman spectroscopy data. Raman spectrometer reproducibility and calibration. [file 1477-3155-5-8-S3.doc]

Raman spectrometer stability experiments employed aqueous SWNT dispersions prepared in TritonX-100 (TrX-SWNTs). There were no major differences between the nanotube resonances observed in TrX-SWNT dispersions (Figure S3) relative to those observed with DM-SWNT dispersions (Figure 4); specifically, the peak shapes of the two RBMs at 281 and 301 cm^-1^ and the G-band at 1590 cm^-1^ were essentially identical. The Raman spectra acquired from four different regions of a TrX-SWNT dispersion are shown in Figure S3. The relative standard deviation (RSD) of G-band peak intensities acquired from the four regions was <10% indicating that the distribution of SWNTs in a TrX-SWNT dispersion was relatively homogeneous. The RSD of G-band peak intensities acquired from the same region of a TrX-SWNT dispersion was <1% (not shown).

**Figure S3**

Raman spectra acquired from four different regions (each separated by ~8 mm) of a 35-mm imaging dish containing a CoMoCAT TrX-SWNT dispersion (10-min probe sonication and two 2-min centrifugations) that was diluted 50.0% (v/v) with deionized water; all spectra were normalized to the same intensity scale.

G-band peak intensities were also used to determine relative SWNT concentrations. The Raman spectra for a series of TrX-SWNT dispersions that were diluted 50.0%, 25.0%, and 12.5% (v/v) with deionized water are shown in Figure S4-top; the plot of G-band peak intensity versus relative SWNT concentration was linear with a correlation coefficient of 0.982 (Figure S5). The absorption spectra for the same series of TrX-SWNT dispersions are shown in Figure S4-bottom for validation purposes; the plot of the absorbance at 1012 nm versus relative SWNT concentration was linear with a correlation coefficient of 0.990 (Figure S5).

50.0%

25.0%

12.5%

50.0%

25.0%

12.5%

**Figure S4**

Normalized Raman **(top)** and absorption **(bottom)** spectra acquired from CoMoCAT TrX-SWNT dispersions (10-min probe sonication and two 2-min centrifugations) that were diluted 50.0%, 25.0%, and 12.5% (v/v) with water.

1012 nm

R^2^ = 0.990

1590 cm^-1^

R^2^ = 0.982

**Figure S5**

Plots of absorbance peak intensities at 1012 nm and Raman G-band peak intensities at 1590 cm^-1^ from the spectra of CoMoCAT TrX-SWNT dispersions shown in Figure S4.
