# Supplementary material for: Single-walled carbon nanotube interactions with HeLa cells
Source: J Nanobiotechnology. 2007 Oct 23;5:8. doi: 10.1186/1477-3155-5-8 (PMC2131758; doi:10.1186/1477-3155-5-8)
Supplement: Additional file 4 — Supporting flow cytometry data. Event plots. [file 1477-3155-5-8-S4.doc]

**DA**

**EA**

**MitoSOX™ Red fluorescence**

**MitoSOX™ Red fluorescence**

**MitoSOX™ Red fluorescence**

**MitoSOX™ Red fluorescence**

**MitoSOX™ Red fluorescence**

**Background fluorescence**

**Background fluorescence**

**Background fluorescence**

**Background fluorescence**

**Background fluorescence**

**A**

**B**

**C**

**Figure S6**

Flow cytometry analysis of intracellular MitoSOX™ Red fluorescence from live HeLa cells incubated at 37 °C for 60 h in: **(A)** DMEM/FBS, **(B)** CoMoCAT DM-SWNTs, **(C)** DMEM/FBS + MitoSOX™ Red, **(D)** DM-SWNTs + MitoSOX™ Red, and **(E)** DMEM/FBS + MitoSOX™ Red + H_2_O_2_. For each plot, the *x*- and *y*-axes denote the background fluorescence detected in the 515−545 nm spectral region, and the MitoSOX™ Red fluorescence detected in the 564−606 nm spectral region, respectively.


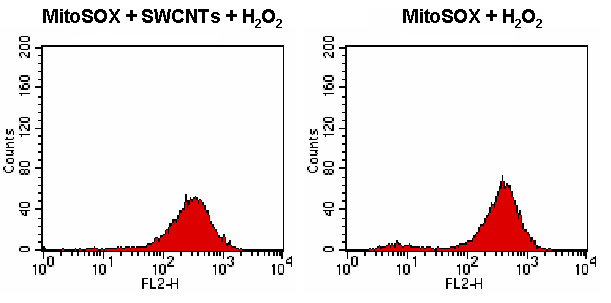


**A**

**B**

**MitoSOX™ Red fluorescence**

**MitoSOX™ Red fluorescence**

**Events**

**Events**

**Figure S7**

Flow cytometry analysis of intracellular MitoSOX™ Red fluorescence from live HeLa cells incubated at 37 °C for 60 h in: **(A)** DMEM/FBS + MitoSOX™ Red + H_2_O_2_ and **(B)** CoMoCAT DM-SWNTs + MitoSOX™ Red + H_2_O_2_. The *x* axis denotes the MitoSOX™ Red fluorescence detected in the 564−606 nm spectral region, and the *y*-axis denotes the number of events recorded for each analysis.
